# A module classification method for light industrial equipment based on improved NSGA2-FCM algorithm

**DOI:** 10.1038/s41598-023-39116-3

**Published:** 2023-08-23

**Authors:** Hui Zheng, Hanwen Guo, Tonglin Pang, Zijian Guo, Xiao Guo

**Affiliations:** 1 School of Economics and Management, Tianjin University of Science and Technology, Tianjin, 300222 China; 2 Lean Management Research Center, Tianjin University of Science and Technology, Tianjin, 300222 China

**Keywords:** Materials science, Techniques and instrumentation, Characterization and analytical techniques, Design, synthesis and processing

## Abstract

In response to the problem that it is easy to fall into local optimum when using the traditional clustering algorithm to divide the modules, this paper improves the initialisation strategy of the NSGA2 algorithm and combines it with the FCM algorithm to propose an improved NSGA2-FCM algorithm for clustering analysis. Firstly, the FBS mapping is used to model the functional structure of the product system and identify the relationship between the product functional structures. Secondly, a correlation synthesis matrix is constructed based on the relationships between the module division drivers. Finally, the improved NSGA2-FCM algorithm is applied to cluster analysis of the product to derive the best module division scheme. The algorithm avoids falling into local optima by optimising the initialisation strategy of the NSGA2 algorithm, while using the FCM algorithm to improve the accuracy of the clustering. This allows the algorithm to explore the solution space more effectively when finding the best module partitioning solution. Finally, the effectiveness of the algorithm for module classification of light industrial equipment is verified using beer fermenters as a case study.

## Introduction

As an effective tool for rapid product design, proper modular classification can improve design efficiency and effectively address the challenges of product quality, manufacturing cycle time, and cost. Most of the current literature on the subject identifies modules by focusing on structural and/or functional aspects, but overlooks modular classification factors. For example, foreign researchers such as Eppinger et al. have studied product modularity development techniques and introduced a matrix-based formal approach for the first time to study modular product structures, indicating correlations between component modules^1^. Rong et al. proposed a product-oriented approach to modular division of the supply chain, establishing a relationship matrix through functional and structural interactions between product components^2^. Ramachandran K et al. investigated the effect of product modularization rate on rapid product launches and found that by locating performance improvements in product modularization, an innovative combination of product architecture, pricing, and timing decisions was achieved for rapid product launches^3^. Wei et al. proposed a predefined set of modules based on assembly and disassembly to generate various working levels or functions for creating multiple product variants and enhancing product utility for design phase decisions through modularity segmentation^4^.

Domestic scholar Jia Yanlin conducted a systematic study on modularity and proposed a general process for modular design of mechanical products, dividing the product modular design process into four parts: modular planning, modular division, module creation, and module combination. Gu Xinxin divided the work of product modularity division into two major parts: modular product platform construction and order product modular design. Zhang Haiyan et al. used the function-principle-behavior-structure design model to map the correlation between system components and applied gap statistics and self-organizing neural network methods for cluster analysis to complete the modular division of special cylindrical gear machine tools, laying the foundation for the reconfigurable research of machine tools^5^. Liu Mingyuan et al. proposed a module classification method based on an improved genetic algorithm. On the premise of obtaining the component design structure matrix and module fitness function, the improved genetic algorithm of difference crossover and neighborhood variation was used to search for the best solution for module classification.

Although previous research on module partitioning has focused more on modularity theory and methods, the sensitivity to module initialization data is low, and there is a lack of exhaustive modularity drivers and metrics, while structured operations also limit other properties of module partitioning.

In this paper, by improving the initialization strategy of the NSGA2 algorithm and combining it with the FCM algorithm, we propose an improved NSGA2-FCM algorithm to analyze clustering results. By changing the value of the cluster center m, we obtain different division schemes to optimize the FCM algorithm, which is not sensitive to the initialization data. This improves the situation where traditional clustering algorithms easily fall into local optima. NSGA2 has good performance in multi-objective optimization problems, while the FCM algorithm effectively performs fuzzy clustering of data samples. By combining the two, multiple optimization objectives (e.g., inter-cluster separation, tightness, etc.) can be considered simultaneously, resulting in better outcomes in clustering problems. In addition, this hybrid approach retains the advantages of the NSGA2 and FCM methods, allowing the algorithm to be more robust and flexible in practical applications. Finally, by taking a beer fermenter as an example, the modular classification of its products is completed, and a modular classification scheme is derived.

## Functional structure modeling of product systems based on FBS mapping

FBS mapping can be considered a design specification process in which a designer transforms a set function into a product that implements that function. The FBS model mapping turns the function into a desired behavior that carries out the function. This desired behavior is employed to select and combine structures in a process called synthesis. During synthesis, the structures generate their own real behavior, allowing the range of desired behaviors to change and the function to be redesigned through them^6^.

**Figure 1:**
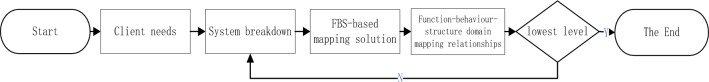
Product modular decomposition process based on FBS mapping.

Products often consist of various functional modules, which are not only interconnected but also possess multiple properties and layers of characteristics within the modules themselves. The approach based on functional-behavioral structures aims to illustrate the connections between product function and structure using a hierarchical analysis of product function to behavior and behavior to structure. To accurately display the relationship between function and structure within the product and reduce the complexity of product analysis, this paper proposes the concept of system level and establishes a product decomposition model of system functional structure. This involves first breaking down the product into several smaller systems and then using the function pointing structure to determine the relationship between product function and structure. The product decomposition process is shown in Fig. 1.

## Improved NSGA2-FCM algorithms

### Improved NSGA2 algorithm

NSGA2 is one of the most efficient and popular evolutionary algorithms for optimization, generating Pareto optimal solutions by analyzing the solution domain. However, the NSGA2 algorithm also suffers from design flaws in its computation, such as the inability to effectively identify pseudo-undominated solutions, low computational efficiency, and poor convergence and distribution of the solution set.

Improving the NSGA2 algorithm primarily involves enhancing the algorithm's initialization strategy, which changes the initial population distribution and improves the algorithm's initial population quality. Since randomly generated initial populations cannot guarantee the validity of the initial scheme, the algorithm may fall into local optima, negatively affecting the optimization performance of the NSGA2 algorithm. To obtain a good initial population, it is crucial for the algorithm to optimally specify an appropriate chromosome size, which is influenced by the number of required modules. Therefore, this paper proposes Eq. (1) for estimating the number of ideal modules and components.

1 $$\mathrm{a}=\sqrt{\mathrm{b}}$$

where a and b are the number of modules and components respectively. ${\text{a}} \ge 2$.^7^

### NSGA2 combined with FCM

(1) FCM algorithm model.

The FCM algorithm minimizes the objective function by calculating the attribute function of the sample points to the class center, which is a function of the degree to which an object x belongs to set A.

Suppose there is a data set ${\text{X,}} {{x_{k}  \in X(k = 1,2,...,n)}}$,and these data is divided into m classes, then there are corresponding m clustering centres, and the affiliation of each sample $\mathop x\nolimits_{k} (k = 1,2,...,n)$ belonging to class $i(i = 1,2,...,c)$ is $\mathop \mu \nolimits_{ik} (0 \le \mathop \mu \nolimits_{ik} \le 1)$. Then the objective function and constraints of the FCM algorithm are shown in Eqs. (2–2)-(2–4).

2 $$ \mathop \mu \nolimits_{ik} \in [0,1](k = 1,2, \cdots ,n;i = 1,2, \cdots ,c) $$

3 $$ \sum\nolimits_{i = 1}^{c} {\mathop \mu \nolimits_{ik} } = 1(\forall k,k = 1,2, \cdots ,n;i = 1,2, \cdots ,c) $$

4 $$ \min \mathop J\nolimits_{m} (U,V) = \sum\nolimits_{k = 1}^{n} {\sum\nolimits_{i = 1}^{c} {\mathop {(\mathop \mu \nolimits_{ik} )}\nolimits^{m} } } \mathop {(\mathop d\nolimits_{ik} )}\nolimits^{2} $$

where the fuzzy clustering matrix $U = [\mathop \mu \nolimits_{ik} ](k = 1,2, \cdots ,n;i = 1,2, \cdots ,c)$ is the set of affiliations and the clustering centre matrix $V = \left\{ {\mathop v\nolimits_{1} ,\mathop v\nolimits_{2} , \cdots ,\mathop v\nolimits_{m} } \right\}$ is the set of m clustering centres; $\mathop d\nolimits_{ik} = \left\| {\mathop x\nolimits_{k} - \mathop v\nolimits_{i} } \right\|$ is the Euclidean distance.

NSGA2-FCM algorithm design.

In most clustering problems, multiple objective functions need to be optimized, such as separation between clusters, tightness, etc. Given these challenges, NSGA2 can achieve satisfactory results in multi-objective optimization problems. Therefore, combining NSGA2 with FCM may offer a better solution for automatically solving fuzzy clustering problems. This hybrid approach preserves the advantages of both NSGA2 and FCM methods, making the system more robust and flexible.

As required by the clustering classification of the FCM algorithm, obtaining the optimal classification result for product modules necessitates that the number of modules be within a reasonable range. If the number of modules is too large, production costs will increase; if the number of modules is too small, the difficulty of product design will increase. This paper is based on a statistical study by Ericsson. The range of module classification, derived from the statistical laws studied by Ericsson et al., is shown in Eq. (5) below.

5 $$ \left[ {\left\| {\sqrt n - l} \right\|,\left\| {\sqrt n + l} \right\|} \right] $$

This puts the module division scheme between the optimal number of where $\left\| {\sqrt n } \right\|$ denotes rounding, $\,\mathrm{l}=1\mathrm{or}\,2.$

The performance of the NSGA2-FCM algorithm is influenced by several parameters, including population size, crossover probability, mutation probability, and the number of iterations. Setting these parameters appropriately is crucial for obtaining high-quality clustering results. Therefore, data testing was employed to find the most suitable parameter settings for the algorithm. In this paper, the population size was set to 50, the crossover probability to 0.8, the mutation probability to 0.05, and the number of iterations to 200.

To implement the NSGA2-FCM algorithm, the operations and data structures of the two algorithms need to be intertwined. In the process, the selection, crossover, and mutation operations of the NSGA2 algorithm are applied to the membership matrix in the FCM algorithm to achieve multi-objective optimization. Specifically, we can treat the membership matrix of the FCM algorithm as the chromosomes of the NSGA2 algorithm and adjust these chromosomes through genetic operations to find the best fuzzy clustering solution.

As a result, the improved NSGA2-FCM algorithm demonstrates better convergence and stability, while being capable of finding solution sets that are close enough to the optimal solution within a limited number of iterations. Furthermore, these solution sets exhibit high consistency across runs.

Improvement of NSGA2-FCM algorithm flow.

**Figure 2:**
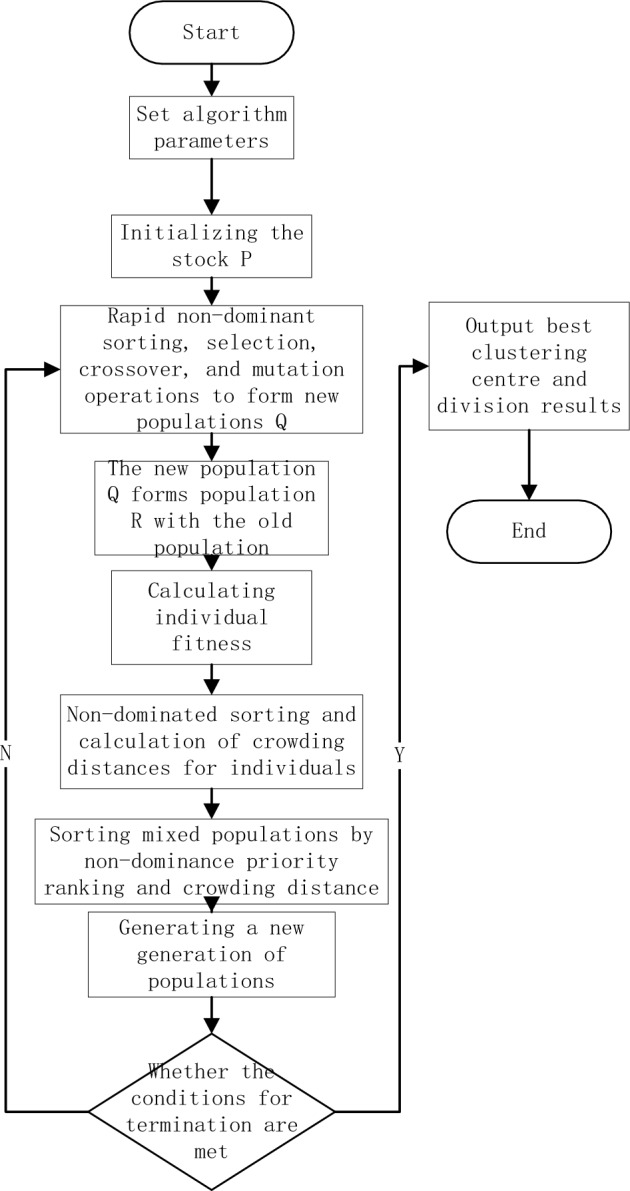
Flow chart of nsga2-fcm clustering algorithm.

The flowchart of the improved NSGA2-FCM algorithm is shown in Fig. 2, with the following steps.

### Comparative performance analysis of algorithms

**Figure 3:**
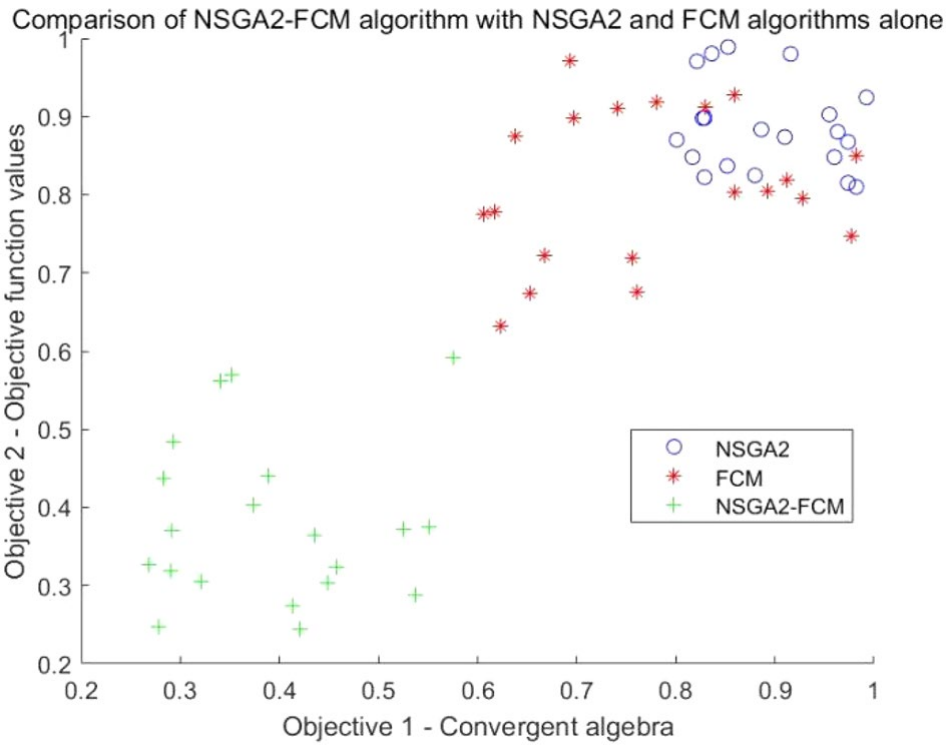
Comparison of optimization results.

To verify the optimization performance of the improved NSGA2-FCM algorithm, its performance was compared with that of the NSGA2 and FCM algorithms alone. The experimental data was simulated and pre-processed, and the results of the comparison are presented in Fig. 3.

As illustrated in Fig. 3, blue represents the results obtained using the NSGA2 algorithm, red represents the results obtained using the FCM algorithm, and green represents the results obtained using the NSGA2-FCM algorithm. The results indicate that the solutions derived from the NSGA2-FCM algorithm perform better on both objective 1 and objective 2, and these solutions are closer to the Pareto optimal solution compared to those achieved by the NSGA2 and FCM algorithms alone. This suggests that the NSGA2-FCM algorithm exhibits superior performance when addressing the light industrial equipment module partitioning problem.

## Creation of the relevant integrated matrix

The weights of the selected modular division factors need to be determined, and the submatrices should be integrated into the corresponding composite matrix using the matrix integration method. This paper employs hierarchical analysis to ascertain the weights of individual module division factors.

**Figure 4:**
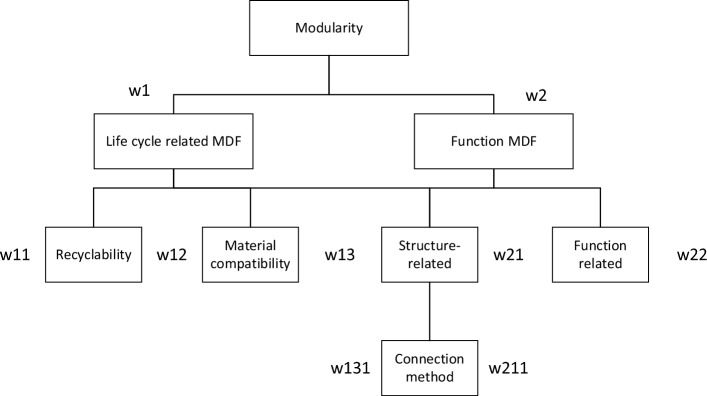
Module division factor weight analysis.

Optimization of the product's functional structure and component recycling are the two primary goals in the product design process. Functional structure optimization involves the modular optimization of product decomposition and the reconfiguration of components into new modules, without reducing the original product's functionality. The recovery optimization objective aims to increase the proportion of product components recovered while maintaining functional optimization. Consequently, the product functional structure objective is as crucial as the recycling objective.Module segmentation drivers are analyzed separately according to the hierarchical criteria of the module segmentation hierarchy analysis presented in Table 1. Figure 4 illustrates the weight analysis of factors involved in module division.

**Table 1:** Hierarchical analysis standard for module division.

Score	Implications
5:5	Factor 1 is equally important than factor 2
6:4	Factor 1 is slightly more important than factor 2
7:3	Factor 1 is more important than factor 2
8:2	Factor 1 is very important than factor 2
9:1	Factor 1 is definitely more important than factor 2

Depending on the percentage divided, the modular division factor should satisfy the following formula:

6 $$ \left\{ \begin{gathered} \mathop w\nolimits_{1} + \mathop w\nolimits_{2} = 1 \hfill \\ \mathop w\nolimits_{11} + \mathop w\nolimits_{12} + \mathop w\nolimits_{13} = \mathop w\nolimits_{1} \hfill \\ \mathop w\nolimits_{131} = \mathop w\nolimits_{13} \hfill \\ \end{gathered} \right. $$

7 $$ \left\{ \begin{gathered} \mathop w\nolimits_{21} + \mathop w\nolimits_{22} = \mathop w\nolimits_{2} \hfill \\ \mathop w\nolimits_{211} = \mathop w\nolimits_{21} \hfill \\ \end{gathered} \right. $$

Establishment of Impact Factor C:

8 $$C=\left\{\begin{array}{l}1, \text{ Two parts have a structural relationship}\\ 0.5\text{ Two parts may have a structural relationship}\\ 0\text{ The two parts are not related}\end{array}\right.$$

Introducing a value of C would:

9 $$ \mathop P\nolimits_{ij} = \mathop w\nolimits_{22} \times \mathop R\nolimits_{f}^{ij} + (\mathop w\nolimits_{131} + \mathop w\nolimits_{211} ) \times \mathop R\nolimits_{g}^{ij} + C \bullet (\mathop w\nolimits_{11} \times \mathop R\nolimits_{d}^{ij} + \mathop w\nolimits_{12} \times \mathop R\nolimits_{m}^{ij} ) $$

The above equation assigns weight values to modularity factors according to their importance to the target.$\mathop P\nolimits_{ij}$ represents the correlation between component i and component j, i.e. the degree of influence of one component on the modularity of another component. From the above equation, the minimum value of the elements in the matrix is 0 and the maximum value is 1. Within this range, the greater the value of the elements, the greater the mutual influence between the components resulting from the combination of different dividing factors, and the more likely it is that two components will be grouped into the same module. Conversely, if the interactions between components under the division factors are smaller, the probability of two components being divided into the same module is correspondingly smaller.

Finally, the relationships between product components are established through a correlation synthesis matrix.$P[n \times n]$ represents the correlation synthesis matrix between components, and $\mathop P\nolimits_{ij}$ is the mutual synthesis between components.

To satisfy later algorithmic optimisation the relevant synthesis matrix has the following properties:

(1) The correlation synthesis matrix is a symmetric matrix, i.e.$P(ij) = P(ji)$.

(2) The correlation synthesis matrix uses the correlations between components and components as matrix elements. So it does not include the component's own relationship value. However, for the simplicity of the algorithm calculation, the component-self relationship value is set to 1.

The relevant combined matrix is then as in equation:

10 $$ P = \left[ {\begin{array}{*{20}c} {\begin{array}{*{20}c} 1 \\ {\mathop P\nolimits_{21} } \\ \end{array} } & {\begin{array}{*{20}c} {\mathop P\nolimits_{21} } \\ 1 \\ \end{array} } & {\begin{array}{*{20}c} \cdots \\ \cdots \\ \end{array} } & {\begin{array}{*{20}c} {\begin{array}{*{20}c} {\mathop P\nolimits_{1(n - 1)} } & {\mathop P\nolimits_{1n} } \\ \end{array} } \\ {\begin{array}{*{20}c} {\mathop P\nolimits_{2(n - 1)} } & {\mathop P\nolimits_{2n} } \\ \end{array} } \\ \end{array} } \\ \vdots & \vdots & \ddots & {\begin{array}{*{20}c} \vdots & \vdots \\ \end{array} } \\ {\mathop P\nolimits_{(n - 1)1} } & {\mathop P\nolimits_{(n - 1)2} } & \cdots & {\begin{array}{*{20}c} 1 & {\mathop P\nolimits_{(n - 1)n} } \\ \end{array} } \\ {\mathop P\nolimits_{n1} } & {\mathop P\nolimits_{n2} } & \cdots & {\begin{array}{*{20}c} {\mathop P\nolimits_{(n - 1)n} } & 1 \\ \end{array} } \\ \end{array} } \right] $$

The correlation matrix is actually a quantification of the interactions between the components of a product and is the basis for modular division using intelligent algorithms. The elements of the correlation matrix are obtained by superimposing the correlations of the various segmentation factors, indicating the total influence of one component on another. The module segmentation method in this paper is a module segmentation method that uses the influence of components to complete the product. The algorithm is designed to optimize the configuration of modular components according to the influence of each component on other components.

## Example analysis

### Structural analysis of beer fermenters

**Figure 5:**
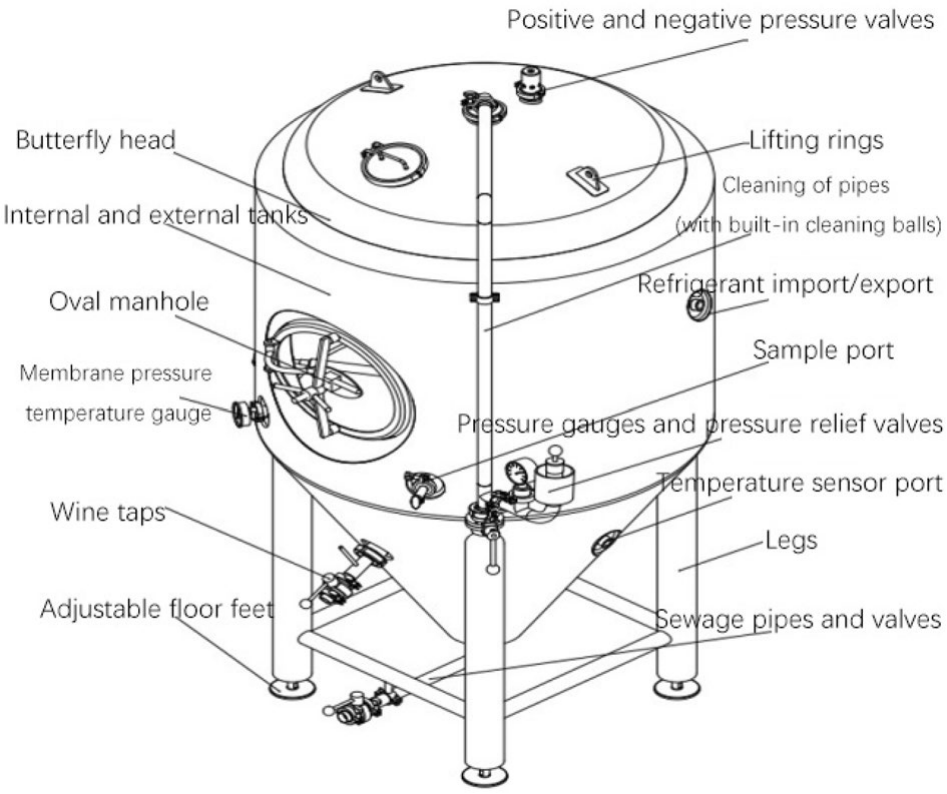
Functional diagram of beer fermentor.

In layman's terms, a beer fermenter is used to produce beer through the fermentation of grain. Its basic structure is shown in Fig. 4:

As can be seen from Fig. 5, the beer fermenter implements the beer fermentation function with the tank, temperature sensor, pressure sensor and cleaning device. With the tank body and stirring device as the core of fermentation and the servo motor as the actuating component, the beer fermentation process is realized by controlling the beer fermenter temperature regulating device and the pressure regulating device. The main components of the beer fermenter are shown in Table 2.

**Table 2:** Main parts of beer fermentor.

No	Name	No	Name
1	Legs	9	Mixers
2	Sampling valve	10	Exhaust valves
3	Discharge valve	11	Pressure gauges
4	Tank body	12	Positive and negative pressure valves
5	Thermometer	13	Cleaners
6	Tank tops	14	Motors
7	Shaft seal	15	Air distributor
8	Couplings	16	Defoamer

It is assumed that the range of beer fermenter products has been determined to be developed according to modular architecture. The specific type of architecture is uncertain, but the number of beer fermenter components is known to be determined. The production of beer fermenter component candidates and the manufacture of composite modules, as well as the decision to assemble, transport and recycle the product, are made by the producer based on the design developed by the designer.

### Modelling the decomposition of beer fermenters

According to the differences in the functions achieved, beer fermenters are mainly divided into fermentation systems, temperature control systems, pressure control systems and power systems.

(1) Fermentation system.

Fermentation system refers to the process of fermenting raw materials in the beer fermenter to produce beer. It includes the tank body, the tank roof, the legs, the sampling valve and the discharge valve. The body and roof components are used to store raw materials to achieve the function of fermenting raw materials and storing semi-finished products. The sampling valve takes samples, the discharge valve discharges semi-finished products and waste when fermentation is complete, and the legs support the fermenter.

(2) Temperature control system.

The temperature system is the fermentation process in the fermenter. The internal temperature rises. This paper only selects the thermometer to achieve the function of displaying the internal temperature of the fermenter.

(3) Pressure control system.

Pressure control system is the fermentation process in which the internal pressure changes. Including pressure gauges, positive and negative pressure valves, exhaust valves, air distributors. Pressure gauge to achieve the function of pressure display, positive and negative pressure valve and exhaust valve to achieve the function of adjusting pressure, air distribution is mainly to control the internal oxygen content of fermenter components.

(4) Power system.

The power system provides power for fermenter mixing and cleaning. Including motor, agitator, cleaner, defoamer, shaft seal, coupling. The motor is the power source, the stirrer is to stir the raw material during the fermentation process to improve the fermentation efficiency, the cleaner is to clean the inside of the fermenter after the fermentation is completed, and the antifoamer is to eliminate the air bubbles generated during the fermentation process to improve the fermentation efficiency. The shaft seal realizes the function of sealing and the coupling plays the role of coupling.

**Figure 6:**
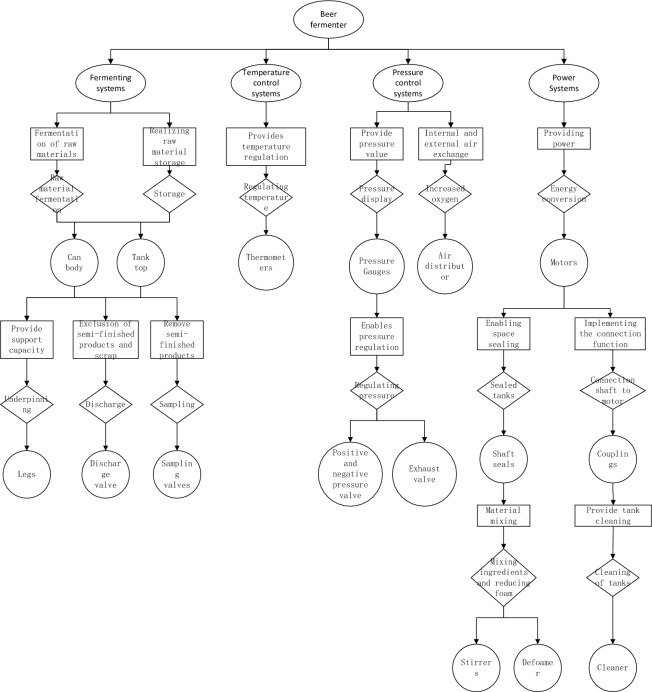
Decomposition results of functional structure of beer fermentor system based on FBS mapping.

Through the above analysis of the beer fermenter, the final product breakdown model shown in Fig. 6 is obtained.

### Division of modules

(1) Establishing the relevant integrated matrix.

According to the module division factors and methods proposed in this paper, the function-related sub-matrices, structure-related sub-matrices, material-related sub-matrices and recyclability-related sub-matrices between beer fermenter modules were evaluated as shown in Tables 3, 4, 5 and 6. According to the characteristics of beer fermenters, the weights of function, structure, material and recyclability are obtained in the order of 36%, 30%, 14% and 20% by hierarchical analysis. The relevant integrated matrix was obtained from Equation as shown in Table 3.

**Table 3:** Function related sub matrix.

NO	1	2	3	4	5	6	7	8	9	10	11	12	13	14	15	16
1	1	0	0	0.7	0	0	0	0	0	0	0	0	0	0	0	0
2	0	1	0.6	0.4	0.2	0	0	0	0	0	0	0	0	0	0	0
3	0	0.6	1	0	0	0	0	0	0	0	0	0	0	0	0	0
4	0.7	0.4	0	1	0.85	0.6	0	0	0	0	0	0	0	0.4	0.6	0.2
5	0	0.2	0	0.85	1	0	0	0	0.4	0	0	0	0.2	0	0	0
6	0	0	0	0.6	0	1	0	0	0	0	0	0	0	0	0	0
7	0	0	0	0	0	0	1	0	0	0	0	0	0	0	0	0
8	0	0	0	0	0	0	0	1	0.8	0	0	0	0.2	0	0	0
9	0	0	0	0	0.4	0	0	0.8	1	0	0	0	0.4	0	0	0
10	0	0	0	0	0	0	0	0	0	1	0.6	0.4	0	0.8	0.2	0.2
11	0	0	0	0	0	0	0	0	0	0.6	1	0.8	0.4	0.3	0	0
12	0	0	0	0	0	0	0	0	0	0.4	0.8	1	0.5	0.8	0.3	0.1
13	0	0	0	0	0.2	0	0	0.2	0.4	0	0.4	0.5	1	0	0	0
14	0	0	0	0.4	0	0	0	0	0	0.8	0.3	0.8	0	1	0.7	0.3
15	0	0	0	0.6	0	0	0	0	0	0.2	0	0.3	0	0.7	1	0.5
16	0	0	0	0.2	0	0	0	0	0	0.2	0	0.1	0	0.3	0.5	1

**Table 4:** Structural correlation submatrix.

NO	1	2	3	4	5	6	7	8	9	10	11	12	13	14	15	16
1	1	0	0.8	0.8	0	0	0	0	0	0	0	0	0	0	0	0
2	0	1	0.4	0.8	0	0	0	0	0	0	0	0	0	0	0	0
3	0.8	0.4	1	0	0	0	0	0	0	0	0	0	0	0	0	0
4	0.8	0.8	0	1	0.3	0	0	0	0	0	0	0	0	0	0	0
5	0	0	0	0.3	1	0	0	0	0	0	0	0	0	0	0	0
6	0	0	0	0	0	1	0.7	0.7	0.3	0.8	0.5	0	0	0	0	0
7	0	0	0	0	0	0.7	1	0	0	0.4	0	0.7	0	0	0	0
8	0	0	0	0	0	0.7	0	1	0.7	0	0	0	0	0	0	0
9	0	0	0	0	0	0.3	0	0.7	1	0	0	0	0	0	0	0
10	0	0	0	0	0	0.8	0.4	0	0	1	0	0	0	0	0	0
11	0	0	0	0	0	0.5	0	0	0	0	1	0.5	0	0	0	0
12	0	0	0	0	0	0	0.7	0.4	0	0	0.5	1	0	0.2	0	0
13	0	0	0	0	0	0	0	0	0	0	0	0	1	0	0	0.2
14	0	0	0	0	0	0	0	0	0	0	0	0.2	0	1	0	0
15	0	0	0	0	0	0	0	0	0	0	0	0	0	0	1	0
16	0	0	0	0	0	0	0	0	0	0	0	0	0.2	0	0	1

**Table 5:** Material correlation submatrix.

NO	1	2	3	4	5	6	7	8	9	10	11	12	13	14	15	16
1	1	0	0	0.8	0	0	0	0	0	0	0	0	0	0	0	0
2	0	1	0	0	0.6	0	0	0	0	0	0	0	0	0	0	0
3	0	0	1	0.8	0	0	0	0	0	0	0	0	0	0	0	0
4	0.8	0	0.8	1	0.8	0.6	0.2	0.4	0.2	0	0	0	0	0.6	0	0.8
5	0	0.6	0	0.8	1	0	0	0	0	0	0	0	0	0	0	0
6	0	0	0	0.6	0	1	0	0	0	0	0	0	0	0	0	0
7	0	0	0	0.2	0	0	1	0	0	0	0	0	0	0	0	0
8	0	0	0	0.4	0	0	0	1	0	0	0	0	0	0	0	0
9	0	0	0	0.2	0	0	0	0	1	0	0	0	0	0	0	0
10	0	0	0	0	0	0	0	0	0	1	0	0.2	0	0	0.8	0.4
11	0	0	0	0	0	0	0	0	0	0	1	0	0	0	0	0
12	0	0	0	0	0	0	0	0	0	0.2	0	1	0.4	0.8	0.6	0
13	0	0	0	0	0	0	0	0	0	0	0	0.4	1	0	0	0
14	0	0	0	0.6	0	0	0	0	0	0	0	0.8	0	1	0.7	0.3
15	0	0	0	0	0	0	0	0	0	0.8	0	0.6	0	0.7	1	0.5
16	0	0	0	0.8	0	0	0	0	0	0.4	0	0	0	0.3	0.5	1

**Table 6:** Recyclability correlation submatrix.

NO	1	2	3	4	5	6	7	8	9	10	11	12	13	14	15	16
1	1	0	0.7	0.8	0.8	0	0	0	0	0	0	0	0	0	0	0
2	0	1	0.4	0	0	0	0	0	0	0	0	0	0	0	0	0
3	0.7	0.4	1	0	0	0	0	0	0	0	0	0	0	0	0	0
4	0.8	0	0	1	0.3	0	0	0	0	0	0	0	0	0	0	0
5	0.8	0	0	0.3	1	0	0	0	0	0	0	0	0	0	0	0
6	0	0	0	0	0	1	0.7	0.7	0.3	0.8	0.7	0	0	0	0	0
7	0	0	0	0	0	0.7	1	0	0	0.4	0	0.7	0	0	0	0
8	0	0	0	0	0	0.7	0	1	0.9	0	0	0.5	0	0	0	0
9	0	0	0	0	0	0.3	0	0.9	1	0	0	0.1	0	0	0	0
10	0	0	0	0	0	0.8	0.4	0	0	1	0	0	0	0	0	0
11	0	0	0	0	0	0.7	0	0	0	0	1	0.7	0	0	0	0
12	0	0	0	0	0	0	0.7	0.5	0.1	0	0.7	1	0	0	0	0
13	0	0	0	0	0	0	0	0	0	0	0	0	1	0	0	0
14	0	0	0	0	0	0	0	0	0	0	0	0	0	1	0	0
15	0	0	0	0	0	0	0	0	0	0	0	0	0	0	1	0
16	0	0	0	0	0	0	0	0	0	0	0	0	0	0	0	1

(2) Performing module classification.

**Figure 7:**
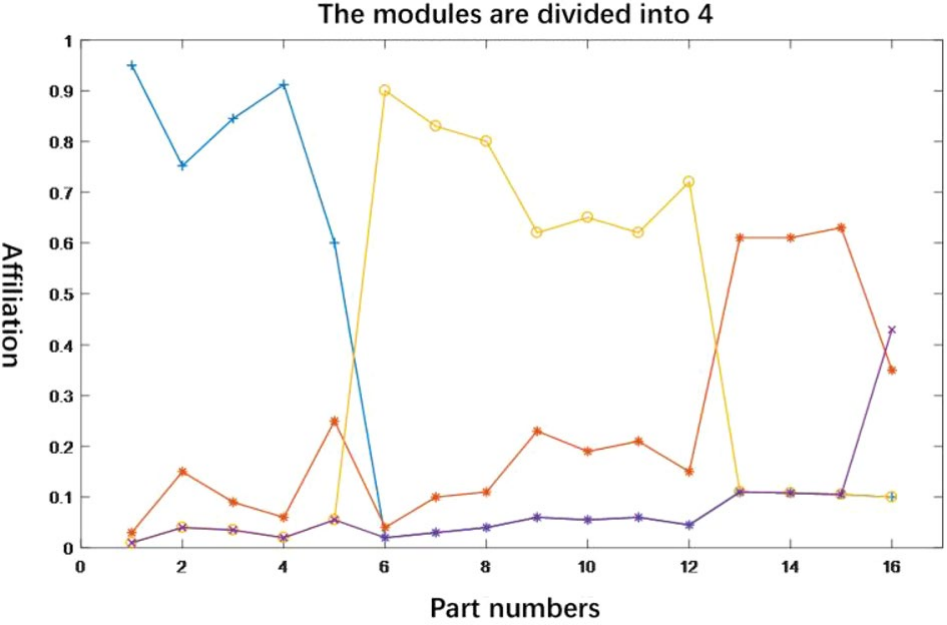
Clustering membership curve with C = 4.

**Figure 8:**
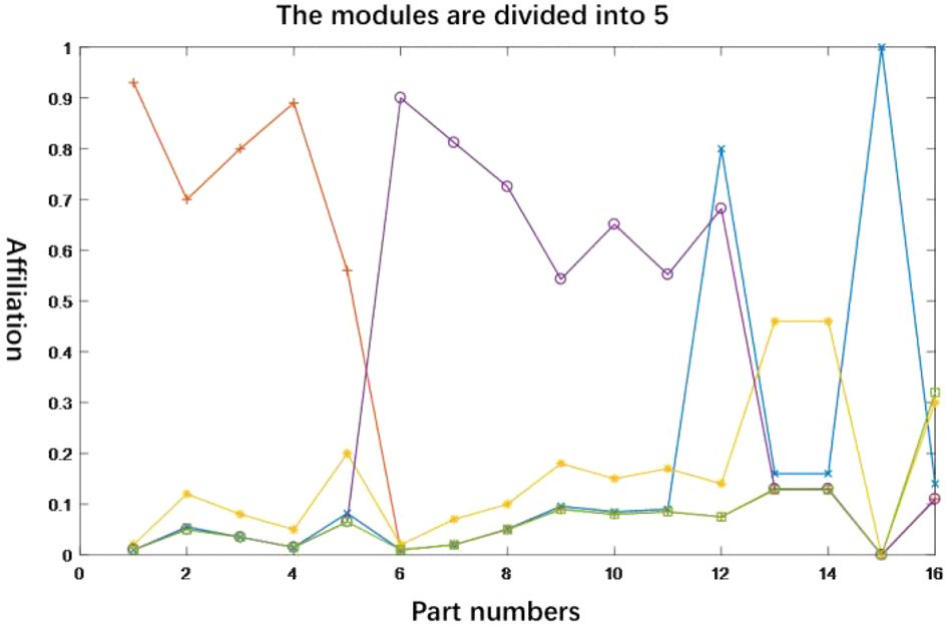
Membership curve of clustering division with C = 5.

**Figure 9:**
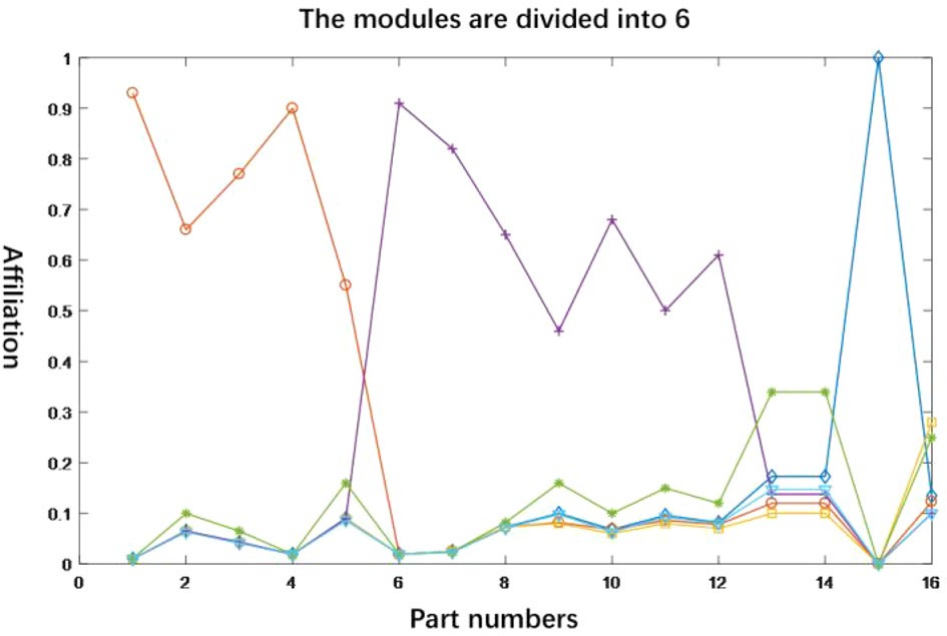
Clustering membership curve of C = 6.

According to Tables 7, the clustering analysis of the components was performed by the modified NSGA2-FCM algorithm. The jQuery parameters were set as follows: population size N is 30, maximum number of iterations 250, crossover probability 0.9, variation probability 0.03, number of clusters m is 3, fuzzy index w is 2, upper bound module 6 and lower bound 0. According to the parameter settings, the algorithm was run 250 times to obtain the affiliation curves of the components and each module, and the results of partitioning clustering of beer fermenter modules are shown in Figs. 7, 8, and 9 respectively.

**Table 7:** Correlation synthesis matrix.

NO	1	2	3	4	5	6	7	8	9	10	11	12	13	14	15	16
1	1	0	0.71	0.8	0.3	0	0	0	0	0	0	0	0	0	0	0
2	0	1	0.63	0.56	0	0	0	0	0	0	0	0	0	0	0	0
3	0.71	0.63	1	0.42	0	0	0	0	0	0	0	0	0	0	0	0
4	0.8	0.56	0.42	1	0.42	0	0	0	0	0	0	0	0	0	0	0
5	0.3	0	0	0.42	1	0	0	0	0	0	0	0	0	0	0	0
6	0	0	0	0	0	1	0.7	0.7	0.42	0.8	0.6	0.16	0	0	0	0
7	0	0	0	0	0	0.7	1	0	0	0.44	0	0.7	0	0	0	0
8	0	0	0	0	0	0.7	0	1	0.83	0	0.04	0.54	0	0	0	0
9	0	0	0	0	0	0.42	0	0.83	1	0	0	0.35	0	0	0	0
10	0	0	0	0	0	0.8	0.44	0	0	1	0	0	0	0	0	0
11	0	0	0	0	0	0.6	0	0.04	0	0	1	0.6	0	0	0	0
12	0	0	0	0	0	0.16	0.7	0.54	0.35	0	0.6	1	0	0.04	0	0
13	0	0	0	0	0	0	0	0	0	0	0	0	1	0.14	0	0
14	0	0	0	0	0	0	0	0	0	0	0	0.04	0.14	1	0	0
15	0	0	0	0	0	0	0	0	0	0	0	0	0	0	1	0
16	0	0	0	0	0	0	0	0	0	0	0	0	0	0	0	1

For different values of the number of clusters, the module partitioning scheme is obtained as shown in Table 8. Among them, it can be seen from Fig. 8 that the module division scheme $\mathop x\nolimits_{1}$ indicates that when the number of modules is 4, components 1, 2, 3 and 4 have higher affiliation in module 1, i.e. module 1 includes components {1, 2, 3, 4}, similarly, module 2 includes components {5, 6, 7}, module 3 includes components {7, 8, 9, 10, 11} and module 4 includes components {12, 13, 14, 15, 16}. Based on the above rules and Fig. 8, the final division scheme can be obtained as shown in Table 8.

**Table 8:** Module division scheme of C = 4–6.

Programme number	No. of modules	Programme of module division
$\mathop x\nolimits_{1}$	4	(1, 2, 3, 4) (5, 6) (7, 8, 9, 10, 11) (12, 13, 14, 15, 16)
$\mathop x\nolimits_{2}$	5	(1, 2, 3) (4, 5, 6) (7, 8, 9) (10, 11, 12) (13, 14, 15, 16)
$\mathop x\nolimits_{3}$	6	(1, 2) (3, 4, 5, 6) (7, 8, 9, 10) (11, 12) (13, 14, 15, 16)

## Conclusions

In this paper, we propose an improved NSGA2-FCM algorithm by combining the NSGA2 initialization strategy with the FCM algorithm. The product functional and structural relationships are identified according to customer requirements and hierarchical models, and a comprehensive numerical matrix is obtained using the hierarchical analysis method for module division factors. A comparative analysis of the algorithm's performance demonstrates that the NSGA2-FCM algorithm outperforms the NSGA2 and FCM algorithms alone in terms of convergence algebra and objective function values, and is closer to the Pareto optimal solution.Using a beer fermenter as an example, a modular partitioning scheme is obtained by employing a combination of the improved NSGA2-FCM algorithm and the related integrated matrix cluster analysis. It is shown that constructing a comprehensive relationship matrix of components based on module partitioning drivers and conducting a comprehensive relationship matrix analysis using the improved NSGA2-FCM algorithm to obtain an optimized modular partitioning scheme improves the issue of the clustering algorithm tending to fall into local optima during partitioning. This approach makes the light industrial equipment module partitioning method more accurate and efficient.

## Data Availability

The datasets generated and/or analysed during the current study are not publicly available due the source of the data is the same as the company I examined, this company does not support the relevant papers to disclose their data for the time being. but are available from the corresponding author on reasonable request.
